# Stigma towards psychiatry: correlating personal experience with existing literature

**DOI:** 10.1192/bjo.2021.365

**Published:** 2021-06-18

**Authors:** Jack Blake, George El-Nimr

**Affiliations:** 1 Final Year Medical Student Keele University School of Medicine; 2 Academic Secretary for the Faculty of Neuropsychiatry at the Royal College of Psychiatrists, Undergraduate Clinical Tutor Keele University School of Medicine, Consultant Neuropsychiatrist North Staffordshire Combined Healthcare NHS Trust

## Abstract

**Aims:**

Stigma towards psychiatry feels rife within medical school and this extends from university life into clinical placements. Mental health remains an unattractive area of medicine and is frequently regarded as subpar by other specialists. Against existing literature, this study compares the authors first hand experiences over the last five years within medical school to evaluate how representative their experiences of stigma in psychiatry are for the wider community and published literature. The study aims to inform the wider discussion on this topic and offer areas where intervention may yield a better perception and hence uptake of this specialism.

**Method:**

Literature review relating to the topic was completed. Studies pertaining to medical students and/or educators views and experiences of psychiatric medical education and clinical placement were included for discussion. A reflection on the first author's specific experiences to date of psychiatry and his intent to pursue psychiatric career was conducted, with careful reference to existing literature. This allowed validating personal experiences in light of shared experience within the medical community in various national and international settings.

**Result:**

Arguably, some non-psychiatric clinicians do inadvertently set the scene early in medical school for the stigma that is to be thrust upon students. This builds upon prospective students ranking psychiatry low for satisfaction, prestige and stating it to be a ‘pseudoscience’ or words to that effect. The lack of understanding from junior medical students of the role of the psychiatrist sees them associating psychosocial education as equivalent to psychiatry. This reinforces the idea of psychiatry being grounded in sciences other than anatomy, biochemistry, physiology and pharmacology. On clinical placement, there is little cross-speciality support for those students who want to be psychiatrists and sometimes even lost opportunities for those publically aspiring towards psychiatry. Placements in psychiatry give students a better understanding of psychiatry but this does not seem to significantly change their career aspirations and this is rather defined from the admission stages.

**Conclusion:**

After comparing experience with literature, stigma towards psychiatry appears to be universal. It may be important to consider the types of students who are being attracted to medical school as currently students seem to have an intrinsic disinterest in psychiatry despite later becoming better informed through psychiatric placement. Culture is notoriously hard to change, particularly within medicine. This stigma exists both in the lay and medical communities with early potentially inaccurate lay views of psychiatry being validated and reinforced throughout medical school.

